# Childhood adversity and autoimmune diseases: global research landscape, immune-related hotspots, and emerging trends from a bibliometric analysis of three databases

**DOI:** 10.3389/fimmu.2026.1864301

**Published:** 2026-08-31

**Authors:** Haisheng Xu, Liuyin Jin, Hanqing Jin, Bin Wang, Xiao Ding

**Affiliations:** 1 Department of Emergency Medicine, Shaoxing Seventh People’s Hospital, Shaoxing, Zhejiang, China; 2 Shaoxing Mental Health Prevention and Treatment Guidance Center, Shaoxing Seventh People’s Hospital, Shaoxing, Zhejiang, China; 3 Department of Science and Education, The Second People’s Hospital of Lishui, Lishui, Zhejiang, China; 4 Neurology and Internal Medicine II, Affiliated Hospital of Shandong University of Traditional Chinese Medicine, Jinan, Shandong, China

**Keywords:** adverse childhood experiences, autoimmune diseases, bibliometric analysis, childhood adversity, immune dysregulation, inflammation, psychoneuroimmunology, research trends

## Abstract

**Background:**

Childhood adversity is increasingly recognized as an important early-life psychosocial exposure that may shape long-term immune function and contribute to autoimmune diseases through chronic stress, neuroendocrine dysregulation, and inflammatory activation. However, the knowledge structure, research hotspots, and developmental trends in this field remain insufficiently characterized.

**Methods:**

Publications on childhood adversity and autoimmune diseases were retrieved from the Web of Science Core Collection, Scopus, and PubMed from database inception to December 31, 2025. After screening, standardization, and deduplication, 319 records were included. Bibliometric analyses were performed using R, VOSviewer, and CiteSpace to evaluate publication trends, contributions of countries, institutions, authors, and journals, collaboration networks, keyword co-occurrence, thematic clustering, and citation impact. Original articles and reviews were reported separately, and an article-only sensitivity analysis was conducted to assess the influence of reviews on the main findings.

**Results:**

Among the 319 publications, 257 (80.6%) were original articles and 62 (19.4%) were reviews. Publication output increased steadily, with rapid growth after 2021 and a peak in 2022. The United States led the field in publication output, citation impact, and international collaboration, while the United Kingdom, Canada, and Germany were also major contributors. Leading institutions included the University of California System, Harvard University, and the University of Manitoba. Major publication venues included *Journal of Psychosomatic Research* and *Psychosomatic Medicine*. Keyword analysis identified childhood adversity, depression, multiple sclerosis, rheumatoid arthritis, stress, autoimmune disease, and inflammation as major hotspots. Highly cited publications indicated that the field is rooted in stress biology, psychoneuroimmunology, inflammatory mechanisms, and disease-specific autoimmune research. Sensitivity analyses showed that major thematic and source-level patterns remained relatively stable after reviews were excluded, whereas reviews were disproportionately represented among highly cited publications.

**Conclusions:**

Research on childhood adversity and autoimmune diseases has developed into a growing interdisciplinary field linking psychosocial stress, immune-inflammatory dysregulation, and disease-specific outcomes. Future research should strengthen standardized definitions, longitudinal and causal evidence, and translational studies on risk stratification and early prevention.

## Graphical Abstract

**Figure:**
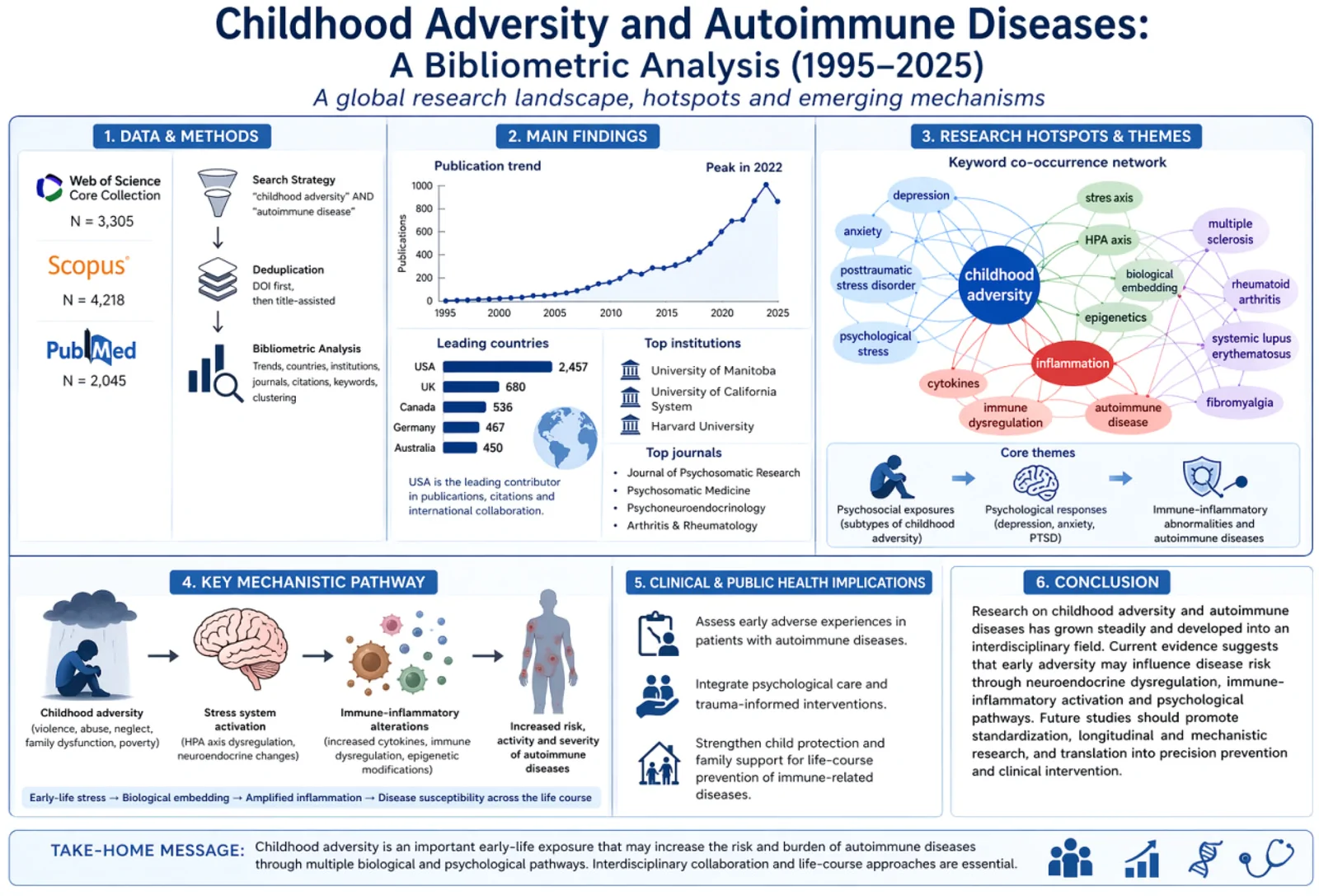

## 1 Introduction

Adverse childhood experiences (ACEs) generally refer to physical, emotional, and sexual abuse, as well as neglect related to household dysfunction, such as parental mental illness, substance abuse, incarceration, domestic violence, and divorce (1). In epidemiological research, ACEs are commonly assessed using a 10-item retrospective questionnaire covering three forms of abuse (emotional, physical, and sexual abuse), two forms of neglect (emotional and physical neglect), and five types of household dysfunction (parental separation or divorce, domestic violence against the mother or stepmother, household substance abuse, household mental illness, and incarceration of a household member) (2). Existing evidence indicates that ACEs are highly prevalent in the general population (3). According to the World Health Organization, 25%–50% of children worldwide have experienced physical abuse, while 20% of girls and 5%–10% of boys have experienced sexual abuse (4). In addition, a 2022 report from the Centers for Disease Control and Prevention indicated that approximately 17% of adults globally reported exposure to at least four different types of ACEs (5). This burden may be even greater in low- and middle-income countries. For example, studies from Bangladesh reported that 99% of surveyed children had experienced physical abuse (6), and 97% had experienced psychological abuse within the family (7). Together, these findings indicate that childhood adversity is highly prevalent and shows substantial variation across socioeconomic contexts.

Importantly, ACEs rarely occur as isolated events. Rather, different forms of childhood adversity, including abuse, neglect, and household dysfunction, often cluster together and exert cumulative and interactive effects across the life course (8). In an adolescent epidemiological study based on the Young HUNT 3 cohort, Rosalie Broekhof and colleagues found that, among 8,199 evaluable adolescents, 65.8% had experienced at least one ACE and 28% had experienced multiple ACEs; household dysfunction was the most common subtype, while the greatest overlap was observed between neglect and abuse (9). This pattern of exposure clustering suggests that the health consequences of childhood adversity are unlikely to be confined to a single outcome or a single biological system. Increasing evidence indicates that early adversity can exert long-term effects on the body through multiple biological pathways, among which immune dysregulation may represent a key mechanism linking childhood adversity to disease risk in adulthood (10). Childhood adversity has been associated not only with hypothalamic-pituitary-adrenal (HPA) axis dysregulation, chronic low-grade inflammation, and epigenetic alterations, but also with a broad range of immune abnormalities, including increased production of proinflammatory cytokines, telomere alterations, heightened susceptibility to illness and infection, reactivation of latent herpesviruses, and impaired antitumor immune responses (11–13). These findings suggest that childhood adversity may increase the risk of persistent physiological dysregulation and disease susceptibility in adulthood by continuously disrupting neuroendocrine-immune homeostasis. Thus, childhood adversity should be understood not only as a psychosocial exposure, but also as an important early-life environmental factor capable of profoundly shaping immune function and biological homeostasis.

Autoimmune diseases comprise a heterogeneous group of chronic immune-mediated disorders characterized by loss of immune tolerance to self-antigens, including rheumatoid arthritis, multiple sclerosis, and systemic lupus erythematosus, among others (14). These disorders are often characterized by chronicity and recurrence, substantially reducing patients’ quality of life and imposing a considerable medical and societal burden. Their etiology is complex and is generally thought to involve the interplay of genetic susceptibility, sex hormones, environmental exposures, infections, and immune dysregulation. In recent years, with advances in immunology and life-course epidemiology, researchers have increasingly recognized that, beyond traditional biomedical risk factors, early-life environmental exposures may also play an important role in the onset and progression of autoimmune diseases (15, 16). A recent systematic review and meta-analysis by Jehanita Jesuthasan and colleagues further showed that exposure to childhood adversity was associated with an increased risk of autoimmune diseases in adulthood, with accumulating evidence observed for conditions such as rheumatoid arthritis, psoriasis, multiple sclerosis, and inflammatory bowel disease (17). Although the existing literature remains limited by substantial heterogeneity, possible publication bias, and variable study quality, current evidence nonetheless suggests that childhood adversity may contribute to the development and progression of immune-mediated chronic diseases through mechanisms such as inflammatory activation, HPA axis dysregulation, disruption of immune tolerance, alterations in the gut microbiota, and prolonged allostatic load (18). However, research in this field remains scattered across multiple disciplines and has largely focused on specific diseases, particular forms of adversity, or single mechanistic pathways, leaving the overall knowledge structure, research hotspots, and evolutionary trends insufficiently characterized.

Although traditional narrative reviews and meta-analyses are valuable for synthesizing evidence on specific scientific questions, they are limited in their ability to systematically depict the overall knowledge structure of a research field, such as publication growth patterns, core authors and institutions, international collaboration networks, highly cited knowledge bases, and the evolution of research hotspots. By contrast, bibliometric methods, combined with quantitative analysis and knowledge-map visualization, can characterize the developmental trajectory and knowledge structure of a field from multiple dimensions. Therefore, conducting a bibliometric study on childhood adversity and autoimmune diseases is of considerable significance. Such an approach can help identify the core knowledge base and landmark publications in this field, reveal the distribution of research productivity and academic collaboration, and detect major research hotspots and emerging directions (19, 20). Accordingly, the present study aimed to perform a systematic bibliometric analysis of research on childhood adversity and autoimmune diseases based on the Web of Science Core Collection (WoSCC), Scopus, and PubMed. By comprehensively evaluating publication trends, major countries and institutions, core authors and journals, citation impact, and keyword co-occurrence patterns, this study sought to map the global research landscape of this field, identify its main research hotspots and evolutionary trends, and provide a reference for future mechanistic, clinical, and translational research.

## 2 Methods

### 2.1 Data sources and search strategy

This study systematically retrieved literature on childhood adversity and autoimmune diseases from three databases: the WoSCC, Scopus, and PubMed (21, 22). The search strategy was constructed using a combination of an “exposure module” and an “outcome module” to maximize topical coverage while minimizing topic contamination. The exposure module mainly included terms such as adverse childhood experiences, childhood adversity, early life adversity, childhood trauma, childhood maltreatment, childhood abuse, as well as terms specifically referring to childhood physical abuse, emotional abuse, sexual abuse, and neglect. The outcome module mainly included representative autoimmune disease terms, such as autoimmune disease(s), autoimmune disorder(s), rheumatoid arthritis, systemic lupus erythematosus, multiple sclerosis, type 1 diabetes, autoimmune thyroiditis, Hashimoto thyroiditis, Graves disease, Sjogren syndrome, and celiac disease. To improve search specificity, broad expressions that might introduce ambiguity regarding life stage, such as sexual abuse alone, were not used directly; instead, more specific terms such as childhood sexual abuse or child sexual abuse were prioritized. The full search strategies are provided in **Supplementary Table 1** (23, 24).

The search period covered database inception to December 31, 2025. Because records from 2026 were still undergoing continuous indexing and dynamic backfilling, directly including them could introduce time-truncation bias and affect the stability of analyses on annual publication trends, collaboration structure, and thematic evolution. Therefore, records from 2026 were excluded from the formal analysis.

### 2.2 Record export and data integration

After retrieval, bibliographic records were exported separately from the three databases. WoSCC records were exported in full-record plain text format, Scopus records in CSV format, and PubMed records in Citation Manager (.nbib) format. All records were then imported into the R 4.5.2 environment and converted into a unified bibliographic data structure using the *bibliometrix* package to support cross-database integrated analysis.

In terms of data processing, records from the three databases were first merged, but automatic deduplication was not performed at the import stage. Instead, year restriction, document type screening, and duplicate identification were carried out sequentially within a unified data-cleaning workflow. This design helped ensure a clear and consistent order of data exclusion across steps, while improving the traceability, verifiability, and reproducibility of the method.

### 2.3 Year restriction, document type screening, and deduplication

Within the merged raw dataset, records published in 2026 were first excluded, together with records with missing publication year information or with publication year that could not be clearly determined. Next, document types were uniformly screened, and only Article and Review records were retained. Original articles and reviews were included to provide a broad overview of both primary research and synthesized evidence in this field. Conference papers, conference abstracts, editorials, letters, news items, corrections, and other non-research records were excluded.

**Figure 1:**
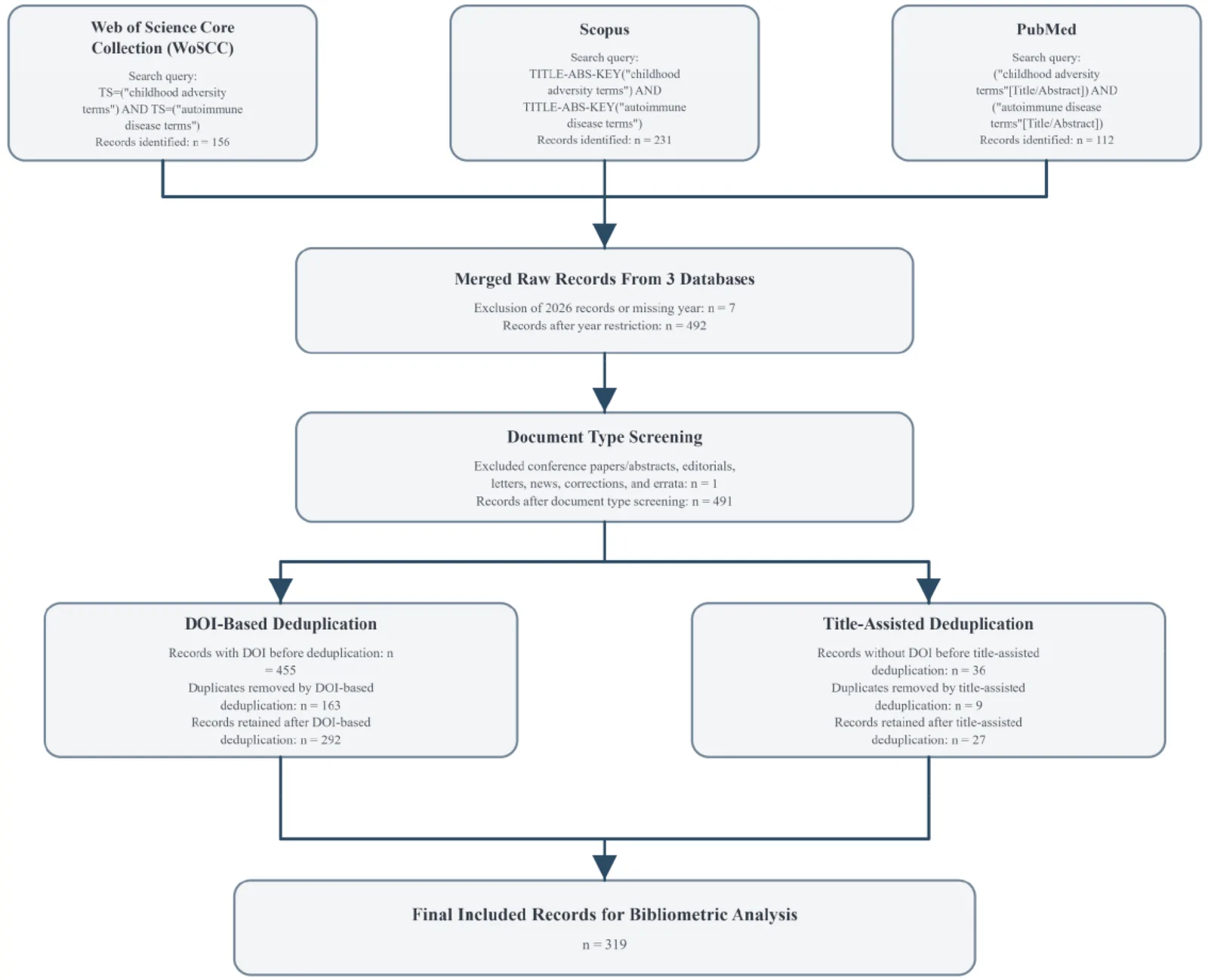
Flowchart of literature retrieval, screening, and deduplication.

Duplicate identification followed a two-step strategy of “DOI-first, title-assisted.” First, DOI fields were standardized by harmonizing letter case and removing DOI prefixes and extra spaces, after which records with DOI information were deduplicated through exact DOI matching. For records without DOI, title-assisted deduplication was further applied: titles were normalized in terms of case, punctuation, and spacing, and duplicate records were identified by combining title information with first author and publication year, thereby improving the accuracy of deduplication among records lacking DOI. The resulting standardized dataset was used for subsequent analyses. The full screening and deduplication workflow is shown in **Figure 1**.

### 2.4 Bibliometric analysis

Based on the cleaned final dataset, bibliometric analyses of research on childhood adversity and autoimmune diseases were conducted from three perspectives: research output, collaboration patterns, and knowledge base. The main analytical components included:

1. annual publication trends, to characterize the developmental trajectory of the field;
2. country/region distribution and international collaboration patterns, to identify major contributing countries and cross-national collaboration features;
3. institutional output and institutional collaboration networks, to identify productive institutions and their collaborative relationships; Before institutional-level analysis, affiliation names were standardized, and department-level entries, institutional variants, and duplicated affiliations within the same publication were checked and merged to avoid repeated counting.
4. author productivity and author collaboration, to identify core author groups;
5. journal distribution and source impact, to identify the major publication venues in this field;
6. highly cited literature and citation performance, to identify the knowledge base and representative studies in this field.

The numbers and proportions of original articles and reviews were reported separately. To assess the potential influence of review articles on the main bibliometric findings, an article-only sensitivity analysis was performed by comparing the overall and article-only datasets with respect to the top 20 keywords and the top 20 journals by publication output.

### 2.5 Keyword co-occurrence and thematic structure analysis

To identify major research themes and characterize the knowledge structure of research on childhood adversity and autoimmune diseases, keyword analyses were performed using R, VOSviewer, and CiteSpace (25). Keyword sources included author keywords (DE), index keywords (ID), and available subject-heading fields (e.g., MH) (26). First, keyword preprocessing was conducted in R using the merged and deduplicated final dataset. This included removing common demographic and methodological stop words, such as human(s), male, female, adult, review, and controlled study, and moderately standardizing major synonyms and closely related expressions. For example, adverse childhood experiences, childhood adversity, early life adversity, childhood trauma, and childhood maltreatment were incorporated into a relatively consistent conceptual framework. Similarly, for disease-outcome-related terms, autoimmune disease(s) and specific disease names were standardized. The cleaned keywords were used for the main-text analyses, whereas the frequencies of the original uncleaned keywords were retained in the Supplementary Materials to enhance result traceability.

The keyword co-occurrence network was constructed in VOSviewer based on document-keyword relationships. The analysis type was set to co-occurrence, and the counting method was full counting. To preserve the core thematic structure while reducing the interference of low-frequency noise on network visualization and cluster interpretation, the minimum keyword occurrence threshold was set at 40. This threshold was not arbitrarily chosen; rather, it was determined after comparing network size, node density, cluster clarity, and graphical readability under different threshold settings. The aim was to highlight stable and interpretable high-frequency themes in the field without overintroducing marginal or incidental terms.

In addition, to identify recent research frontiers and shifts in hotspots from a temporal perspective, keyword co-occurrence analyses were further performed in CiteSpace. To balance the depiction of long-term thematic evolution and the identification of recent frontiers, two time windows were used in CiteSpace: first, the full time span from the publication of the earliest relevant article to December 31, 2025, to display the overall evolutionary trajectory of research themes; second, the most recent 10-year window (2015-2025), to increase sensitivity to recent hotspot shifts and frontier changes. This additional 10-year subwindow was included because publication growth became more pronounced and thematic structures more active during this period, whereas early publications were relatively sparse and could weaken the visibility of recent developments if included within a single evolutionary view. The time slice was set at 1 year per slice. Term sources included titles, abstracts, author keywords (DE), and keywords plus (ID). Network link strength was calculated using cosine, and the node selection criterion was the g-index (k = 25) (27).

### 2.6 Institutional collaboration network analysis

Institutional collaboration network analysis and journal density distribution analysis were both performed in VOSviewer. For the institutional collaboration network, the analysis type was set to *Co-authorship*, the unit of analysis was *Organizations*, and the counting method was *full counting*. To balance completeness and interpretability of the network structure, the inclusion thresholds were set as follows: a minimum of 20 publications and a minimum of 0 citations. This threshold selection was based on the following considerations: if the threshold were too low, a large number of low-output and weakly connected institutions would be introduced, resulting in an overly dense network and increased visual noise; if the threshold were too high, institutions with meaningful bridging roles but slightly lower output might be omitted. In addition, to reduce the amplifying effect of very large multi-institutional papers on network centrality and clustering structure, the option *Ignore documents co-authored by a large number of organizations* was selected, and the *Maximum number of organizations per document* was set to 25. This parameter setting helped preserve major collaborative relationships while reducing the disproportionate influence of a small number of mega-collaboration papers, thereby improving the stability and interpretability of the institutional collaboration results.

For journal distribution analysis, the analysis type was set to *Bibliographic coupling*, the unit of analysis was *Sources*, and the counting method was also *full counting*. To balance the stable identification of core journals and the readability of the visualization, the journal inclusion thresholds were set at a minimum of 3 publications and a minimum of 0 citations. Under this threshold, 215 source journals entered the initial candidate set, of which 15 met the threshold and were included in the final analysis. This setting helped exclude peripheral journals with very low publication output and weak connections, thereby reducing the interference of low-frequency nodes with network structure and visual presentation, while retaining the main representative publication sources in this field. For visualization, a network was constructed based on bibliographic coupling relationships among journals, and a journal density map was further generated using *Density Visualization*. Areas with higher color density indicate greater connection strength and clustering intensity among journals, suggesting stronger knowledge relatedness and publication activity in those areas. This analysis helped identify core journal clusters and the main knowledge dissemination platforms in research on childhood adversity and autoimmune diseases.

### 2.7 Citation relationship and citation performance analysis

Citation relationship analysis depends on complete, structurally consistent, and comparable reference metadata. Considering the differences among databases in citation coverage, reference-field structure, and counting rules, and particularly the fact that PubMed does not provide cited-reference data with the same level of structure as WoSCC, citation relationship analysis in this study was conducted primarily based on WoSCC data in order to reduce bias arising from citation incompleteness and database heterogeneity.

Based on WoSCC data, two main citation indicators were examined: (1) Global Citations, defined as the total number of times a publication was cited by all records indexed in the WoSCC database, reflecting its overall influence across the broader academic literature; and (2) Local Citations, defined as the number of times a publication was cited by other publications within the dataset included in this study, reflecting its internal knowledge influence and hub position within the specific field of childhood adversity and autoimmune diseases. On this basis, the top 10 publications ranked by Global Citations and the top 10 ranked by Local Citations were identified and reported separately, in order to capture key studies with both broad academic influence and core internal importance within the field. In addition, to assess the distribution of academic influence at a more macro level, total citation counts were further calculated and presented for journals, countries, and authors, and the top 10 journals, countries, and authors ranked by total citations were compared.

For citation-based sensitivity analysis, the top 20 WoSCC publications ranked by Global Citations in the complete Article-and-Review dataset were compared with those in the article-only dataset, and the proportion of review articles among the top-cited publications was also examined.

In summary, this study adopted a modular analytical strategy: the integrated multi-database dataset was used to describe the field’s academic output pattern, collaboration modes, and thematic structure, whereas citation relationship analyses were conducted primarily on WoSCC data. This strategy aimed to maximize coverage while also ensuring structural consistency for citation analysis and improving the reliability of result interpretation.

## 3 Results

### 3.1 Literature screening and inclusion for studies on childhood adversity and autoimmune diseases

A total of 499 records were initially retrieved from the three databases, including 156 from the Web of Science Core Collection (WoSCC), 231 from Scopus, and 112 from PubMed. After applying the publication year restriction, 7 records published in 2026 or with missing publication year information were excluded, leaving 492 records. Following document type screening, 1 non-Article/Review record was removed, and 491 records were retained for deduplication. During the DOI-based deduplication stage, 163 duplicates were removed from the 455 records with DOI information, leaving 292 records. For the 36 records without DOI information, title-assisted deduplication was further performed, resulting in the exclusion of 9 duplicates and the retention of 27 records. After merging these two subsets, a total of 319 publications were ultimately included in the subsequent bibliometric analyses. Among the final 319 publications, 257 (80.6%) were original articles and 62 (19.4%) were reviews.

### 3.2 Annual publication trends in research on childhood adversity and autoimmune diseases

**Figure 2:**
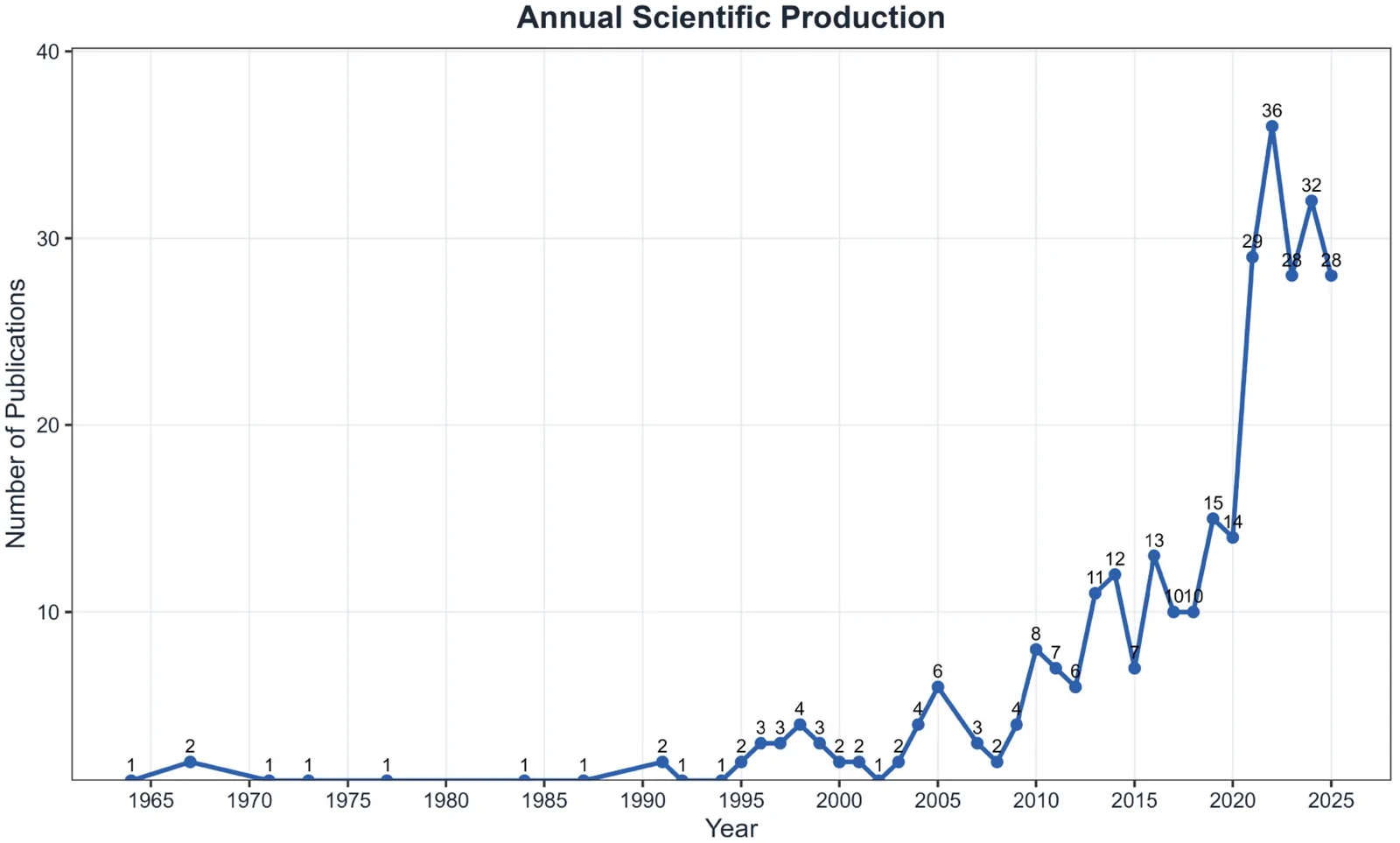
Annual publication trend of research.

**Figure 2** illustrates the annual number of publications in this field. Overall, the annual publication output showed a steady upward trend, although the growth rate varied markedly across different periods. In the early stage (1964–1993), publications were sporadic, with only 1–2 papers published in most years, suggesting that this topic remained at an exploratory stage for a considerable period. Between 1994 and 2008, the annual number of publications began to increase slowly with slight fluctuations, but generally remained at a low level, usually not exceeding six papers per year. This indicates that the field gradually attracted scholarly attention but had not yet developed sustained research momentum. Since 2009, the number of publications has entered a more pronounced growth phase, and since 2013 it has generally remained at a relatively high level. Between 2021 and 2025, publication output increased substantially, reaching 29, 36, 28, 32, and 28 papers, respectively, with the annual peak occurring in 2022 (36 papers). Taken together, these findings indicate that research in this area has expanded rapidly in recent years, and that the association between childhood adversity and related disease outcomes has become an increasingly active and sustained topic of academic inquiry.

### 3.3 Country distribution and collaboration patterns

**Table 1** presents the top 30 countries in terms of publication output and their patterns of international collaboration in research on childhood adversity and autoimmune diseases. Overall, the United States occupied an overwhelmingly dominant position in this field, ranking first with 202 publications, far exceeding all other countries. It also had 146 multiple-country publications, with an MCP% of 72.3%, indicating that it was not only the largest knowledge producer but also the central hub in the international collaboration network. The United Kingdom, Canada, and Germany ranked second to fourth with 38, 33, and 23 publications, respectively, and their MCP% values were 57.9%, 60.6%, and 65.2%, suggesting that these countries also had strong research productivity and active cross-national collaboration. Among countries with relatively high publication output, Australia published 17 papers but had an MCP% as high as 94.1%, while Denmark and Switzerland reached 88.9% and 85.7%, respectively, indicating that research production in these countries relied heavily on international collaboration. Sweden, France, Italy, and Norway also showed moderate to high output, with MCP% values mostly ranging from 58% to 75%, reflecting the generally strong cross-national collaborative characteristics of European countries in this field. In contrast, Brazil, India, and Romania had MCP% values of 23.1%, 44.4%, and 33.3%, respectively, while Turkey produced only single-country publications (MCP% = 0), suggesting that research output in some countries was generated more independently. In addition, several countries with relatively limited publication output, such as Mexico, Finland, Iceland, Luxembourg, Thailand, and South Korea, all had MCP% values of 100.0%, meaning that all papers included in the analysis were produced through international collaboration. Overall, the field shows a global research landscape centered on the United States, with additional support from countries such as the United Kingdom and Canada, while international collaboration plays an important role in research production across most countries.

**Table 1:** Top 30 countries in childhood adversity and autoimmune disease research.

Rank	Country	Publications	SCP	MCP	MCP percent
1	United States	202	56	146	72.3
2	United Kingdom	38	16	22	57.9
3	Canada	33	13	20	60.6
4	Germany	23	8	15	65.2
5	Australia	17	1	16	94.1
6	Brazil	13	10	3	23.1
7	Sweden	12	3	9	75.0
8	France	12	5	7	58.3
9	Italy	12	5	7	58.3
10	Norway	11	4	7	63.6
11	Denmark	9	1	8	88.9
12	Spain	9	4	5	55.6
13	India	9	5	4	44.4
14	Switzerland	7	1	6	85.7
15	China	7	3	4	57.1
16	Israel	7	3	4	57.1
17	Greece	6	3	3	50.0
18	Romania	6	4	2	33.3
19	Turkey	5	5	0	0.0
20	Mexico	4	0	4	100.0
21	Netherlands	4	1	3	75.0
22	South Africa	4	2	2	50.0
23	Finland	3	0	3	100.0
24	Iceland	3	0	3	100.0
25	Luxembourg	3	0	3	100.0
26	Thailand	3	0	3	100.0
27	Belgium	3	1	2	66.7
28	South Korea	2	0	2	100.0
29	Pakistan	2	1	1	50.0
30	Poland	2	1	1	50.0

SCP, single-country publications; MCP, multiple-country publications.

### 3.4 Top 10 authors, journals, and institutions in research on childhood adversity and autoimmune diseases

**Figure 3:**
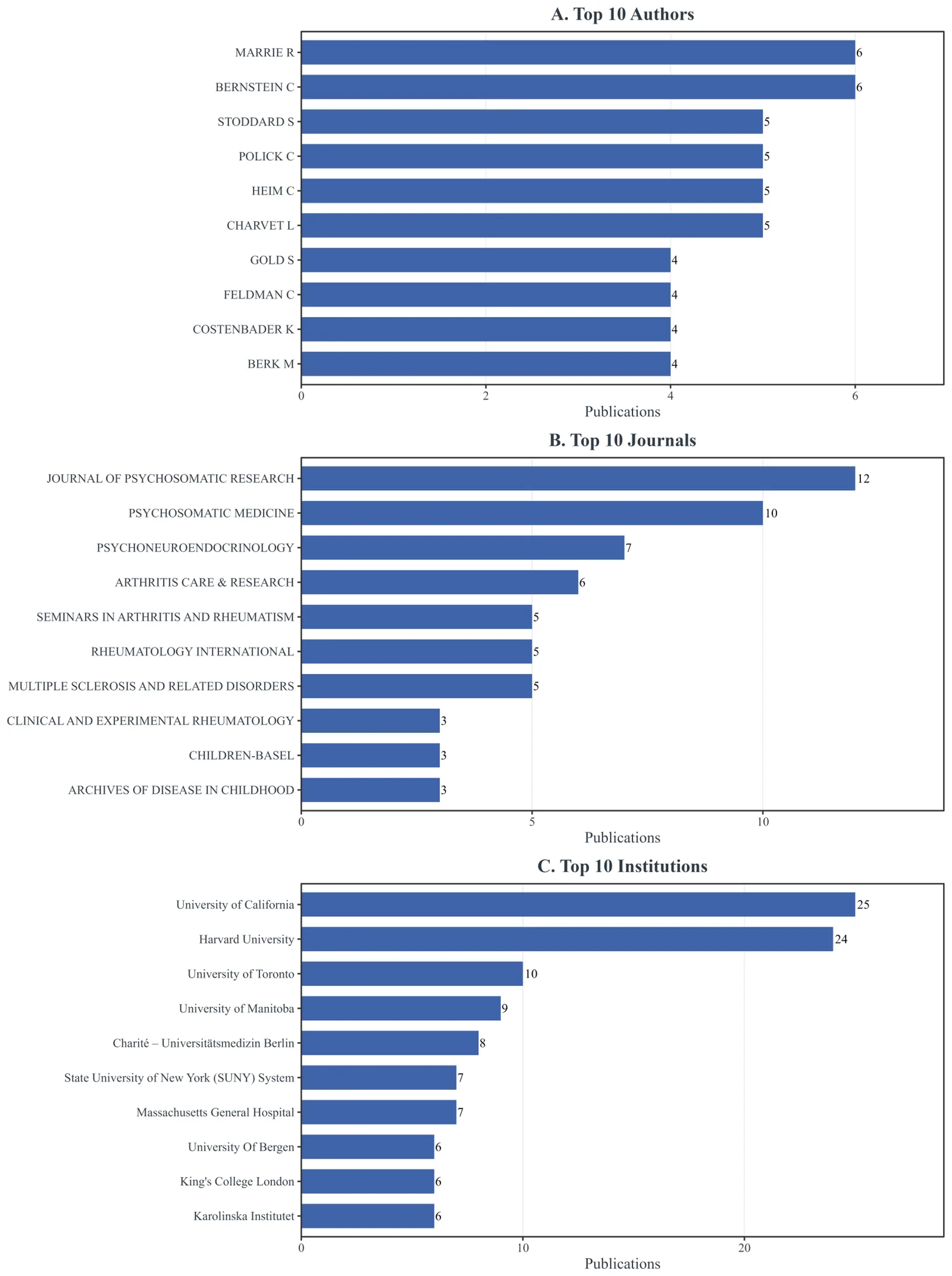
Top 10 authors, journals, and institutions by publication output. **(A)** Top 10 authors based on publication count. **(B)** Top 10 journals by the number of publications. **(C)** Top 10 institutions with the highest publication output.

**Figure 3** presents the top 10 authors, journals, and institutions in the field of childhood adversity and autoimmune diseases. In terms of publication output, a relatively stable knowledge production structure has begun to emerge across authors, journals, and institutions.

At the author level (**Figure 3A**), Marrie R and Bernstein C ranked first jointly, with six publications each. Stoddard S, Polick C, Heim C, and Charvet L each published five papers, forming the second tier. Gold S, Feldman C, Costenbader K, and Berk M each contributed four publications. Overall, the differences in publication counts among leading authors were relatively small, suggesting that the field has not yet been dominated by a single highly prolific scholar, but instead shows a pattern of activity shared by multiple researchers.

At the journal level (**Figure 3B**), *Journal of Psychosomatic Research* ranked first with 12 publications, followed by *Psychosomatic Medicine* with 10 publications. *Psychoneuroendocrinology* published 7 papers, and *Arthritis Care & Research* published 6 papers. In addition, *Seminars in Arthritis and Rheumatism*, *Rheumatology International*, and *Multiple Sclerosis and Related Disorders* each published 5 papers. This distribution indicates that research outputs in this field are mainly concentrated in journals related to psychosomatic medicine, psychoneuroendocrinology, and rheumatology/immunology, reflecting a clear interdisciplinary character.

After institutional name standardization and deduplication, the institutional-level analysis showed that the University of California ranked first with 25 publications, followed by Harvard University with 24 publications. The University of Toronto ranked third with 10 publications, followed by the University of Manitoba with 9 publications and Charité–Universitätsmedizin Berlin with 8 publications. The State University of New York (SUNY) System and Massachusetts General Hospital each contributed 7 publications, while the University of Bergen, King’s College London, and Karolinska Institutet each contributed 6 publications. Overall, the most productive institutions were mainly concentrated in North America and Europe, suggesting an evident regional clustering of research capacity in this field.

### 3.5 Keyword co-occurrence and distribution of research hotspots

**Figure 4:**
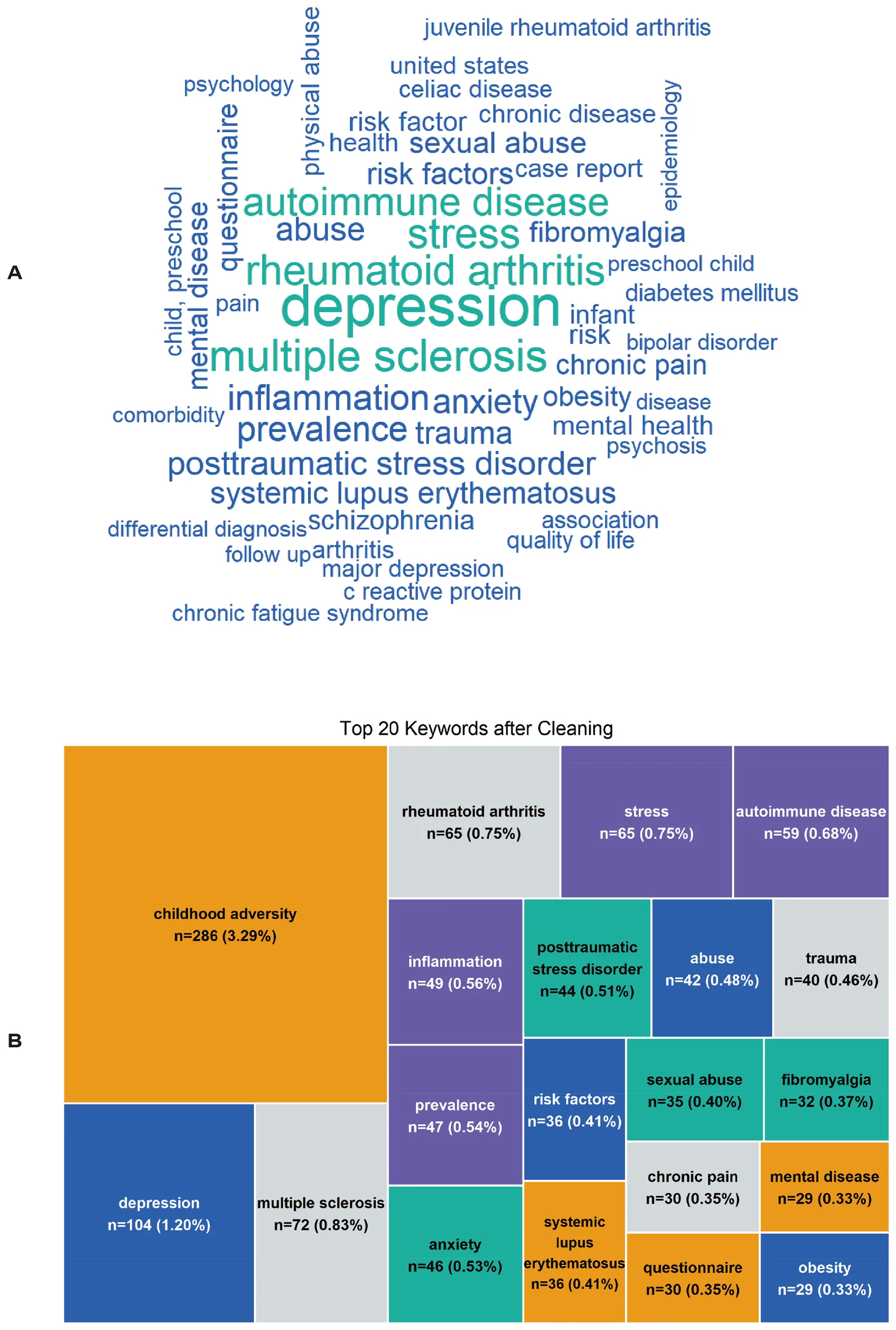
Keyword distribution after cleaning. **(A)** Word cloud of keywords. **(B)** Treemap of the top 20 keywords after cleaning. Colors are used only for visual distinction among keyword blocks, whereas block size represents keyword frequency.

**Figure 4** presents the keyword analysis of research on childhood adversity and autoimmune diseases and illustrates the overall distribution of cleaned keywords and the composition of high-frequency themes. **Figure 4A** shows a keyword word cloud, in which word size reflects frequency of occurrence. The results indicate that childhood adversity was the most central keyword, followed by depression, multiple sclerosis, rheumatoid arthritis, stress, and autoimmune disease, suggesting that research in this field mainly focuses on the relationships between childhood adversity and mental health outcomes, autoimmune diseases, and inflammation-related conditions. At the same time, keywords such as posttraumatic stress disorder, inflammation, anxiety, fibromyalgia, systemic lupus erythematosus, chronic pain, and obesity also appeared frequently, indicating that the research themes have gradually expanded toward stress responses, inflammatory mechanisms, and a range of specific disease outcomes.

**Figure 4B** shows a treemap of the top 20 high-frequency cleaned keywords, further illustrating their relative proportions. Among them, childhood adversity had the highest frequency (n = 286, 3.29%), followed by depression (n = 104, 1.20%), multiple sclerosis (n = 72, 0.83%), rheumatoid arthritis (n = 65, 0.75%), and stress (n = 65, 0.75%). In addition, autoimmune disease, inflammation, prevalence, anxiety, and posttraumatic stress disorder also accounted for substantial proportions, indicating that the field has formed a research pattern centered on childhood adversity as the key exposure, with mental health problems and immune-inflammatory diseases as the major outcomes.

### 3.6 Institution collaboration network, keyword co-occurrence network, and journal density visualization based on VOSviewer

**Figure 5:**
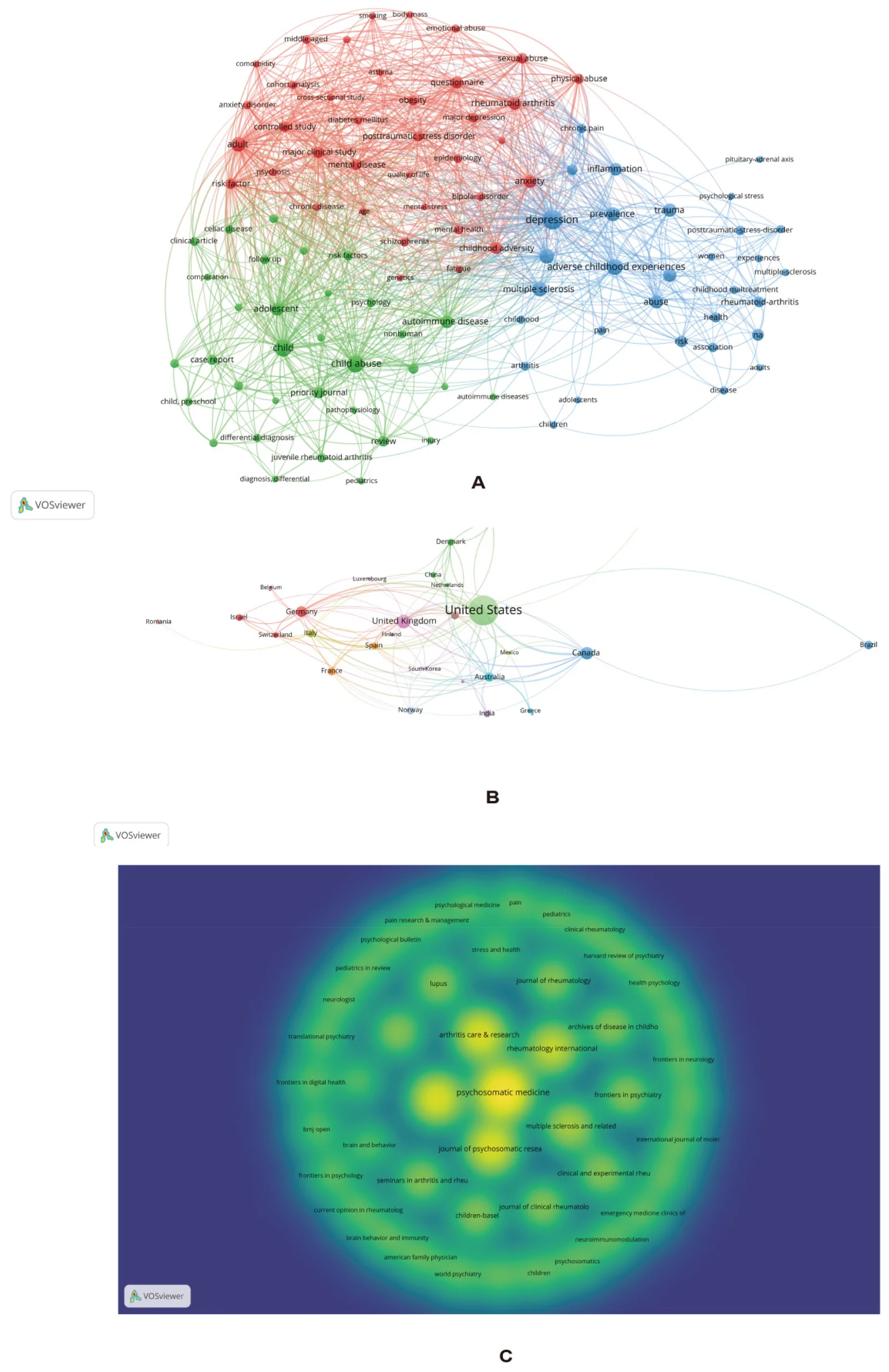
Knowledge structure and collaboration patterns in research on childhood adversity and autoimmune diseases. **(A)** Keyword co-occurrence network. Colors indicate keyword clusters identified by VOSviewer based on co-occurrence relationships. **(B)** Country collaboration network. Colors indicate keyword clusters identified by VOSviewer based on co-occurrence relationships. **(C)** Journal density map.

**Figure 5** presents the knowledge structure and academic landscape of research on childhood adversity and autoimmune diseases from three perspectives: keywords, country collaboration, and journal distribution. **Figure 5A** shows the keyword co-occurrence network, indicating that relatively clear thematic clusters have emerged in this field. The core high-frequency keywords included adverse childhood experiences, depression, multiple sclerosis, rheumatoid arthritis, abuse, inflammation, and trauma, suggesting that the main research focus lies in the associations among childhood adversity exposure, mental health outcomes, inflammatory responses, and representative autoimmune diseases. **Figure 5B** presents the country collaboration network. The United States occupied the central position, with the largest node and the densest connections, and maintained close collaborative ties with Canada, the United Kingdom, Australia, China, and other countries. This indicates that international collaboration in this field is led mainly by North American and European countries, while also showing certain cross-regional cooperative characteristics. **Figure 5C** shows the journal density visualization, with hotspots concentrated in journals such as *Psychosomatic Medicine*, *Journal of Psychosomatic Research*, *Arthritis Care & Research*, and *Multiple Sclerosis and Related Disorders*. This suggests that the topic is mainly distributed across journals in psychosomatic medicine, rheumatology/immunology, neuroimmunology, and related interdisciplinary areas.

### 3.7 Keyword clustering in CiteSpace

**Figure 6:**
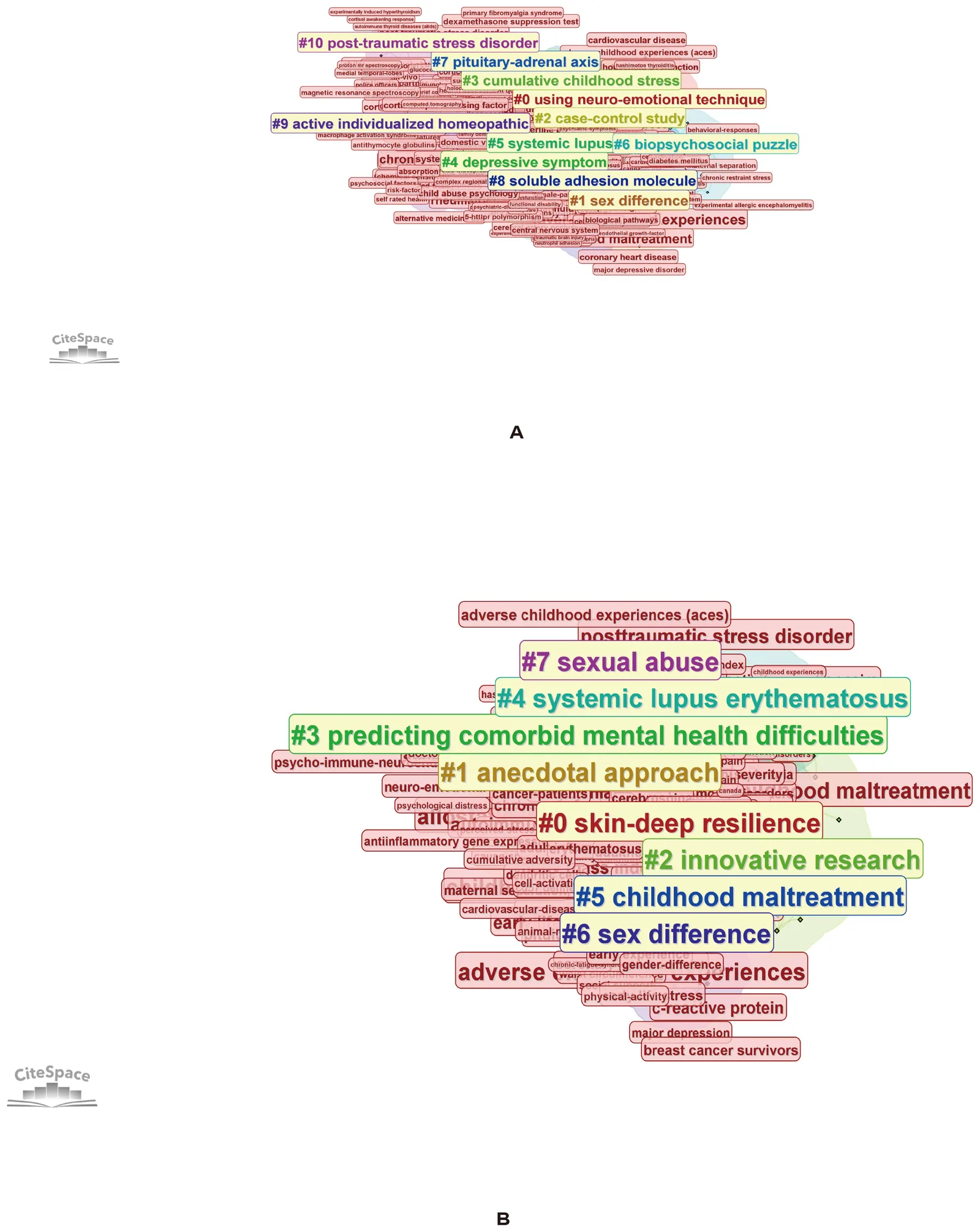
Keyword clusters identified by CiteSpace. **(A)** 2015–2025; **(B)** from inception to 2025.

**Figure 6A** and 6B present the keyword clustering structures generated by CiteSpace under different time windows. **Figure 6A** shows the clustering results for the recent 10 years (2015–2025), whereas **Figure 6B** shows the clustering results for the entire study period from the first relevant publication to 2025. Overall, both time windows indicate that the field has developed a relatively stable research framework centered on childhood adversity exposure, mental health outcomes, immune-inflammatory mechanisms, and specific chronic disease outcomes. However, compared with the full-period analysis, the clusters in the recent decade were more concentrated and showed a stronger emphasis on recently emerging research directions, particularly immune-inflammatory mechanisms and specific disease-related outcomes.

From the results of the recent 10-year window (**Figure 6A**), research hotspots were mainly concentrated on themes such as post-traumatic stress disorder, cumulative childhood stress, pituitary-adrenal axis, depressive symptom, systemic lupus, sex difference, and soluble adhesion molecule. These findings suggest that recent studies have continued to focus on the association between childhood adversity and mental disorders, while also extending toward neuroendocrine mechanisms, inflammatory responses, and immune-related pathways, as well as toward specific disease outcomes such as systemic lupus erythematosus and cardiovascular disease, reflecting a strong orientation toward mechanistic exploration and clinical translation.

From the full-period results (**Figure 6B**), the clustering themes covered a broader range, including adverse childhood experiences, childhood maltreatment, sexual abuse, sex difference, systemic lupus erythematosus, predicting comorbid mental health difficulties, and skin-deep resilience. Compared with the recent 10-year window, the full-period clustering more clearly reflects that the knowledge base of this field was initially built on core exposure concepts such as ACEs, child maltreatment, and sexual abuse, and then gradually extended to issues such as comorbid mental health problems, disease susceptibility, and resilience.

Taken together, the thematic evolution of this field appears to have progressed from early identification of the core exposure types of childhood adversity and their health consequences to more recent in-depth exploration of mechanistic pathways, sex differences, psychiatric comorbidities, and specific chronic disease outcomes. This suggests that the research focus is shifting from descriptive association studies toward mechanism-oriented and precision intervention-oriented research.

### 3.8 Global citations and local citations

**Tables 2** and **3** present the top 10 representative publications ranked by global citations and local citations, respectively, among the studies included in this analysis. In terms of global citation performance, highly cited publications mainly focused on stress, inflammation, depression, and the relationship between childhood trauma and mental and physical diseases. Among them, the article by Slavich GM published in *Psychological Bulletin* in 2014, *From stress to inflammation and major depressive disorder: a social signal transduction theory of depression*, had the highest number of global citations (1,633), indicating its substantial influence in the broader academic field (28). The second most globally cited publication was Heim C’s 2000 study published in *Psychoneuroendocrinology* on hypocortisolism and the mechanisms linking stress-related bodily disorders (1,331 citations) (29). The article *Cumulative childhood stress and autoimmune diseases in adults*, published by Dube SR in *Psychosomatic Medicine* in 2009, ranked third (509 citations), demonstrating that research on the association between cumulative childhood stress and autoimmune diseases has become an important part of the knowledge base in this field (30). Other highly cited studies addressed bipolar disorder, fibromyalgia, post-traumatic stress disorder, and the long-term health consequences of childhood maltreatment, highlighting the strong intersection of psychiatry, immune-inflammatory mechanisms, and chronic disease research in this area.

**Table 2:** Top 10 most globally cited publications.

No.	First author	Article title	Journal	Year	Global citations
1	SLAVICH GM	From stress to inflammation and major depressive disorder: a social signal transduction theory of depression	PSYCHOL BULL	2014	1633
2	HEIM C	The potential role of hypocortisolism in the pathophysiology of stress-related bodily disorders	PSYCHONEUROENDOCRINO	2000	1331
3	DUBE SR	Cumulative childhood stress and autoimmune diseases in adults	PSYCHOSOM MED	2009	509
4	ROWLAND TA	Epidemiology and risk factors for bipolar disorder	THER ADV PSYCHOPHARM	2018	291
5	GOODWIN RD	Association between childhood trauma and physical disorders among adults in the United States	PSYCHOL MED	2004	285
6	WALKER EA	Psychosocial factors in fibromyalgia compared with rheumatoid arthritis: II. Sexual, physical, and emotional abuse and neglect	PSYCHOSOM MED	1997	233
7	HEIM C	Abuse-related posttraumatic stress disorder and alterations of the hypothalamic-pituitary-adrenal axis in women with chronic pelvic pain	PSYCHOSOM MED	1998	227
8	SLAVICH GM	Assessing Lifetime Stress Exposure Using the Stress and Adversity Inventory for Adults (Adult STRAIN): An Overview and Initial Validation	PSYCHOSOM MED	2018	207
9	SPRINGER KW	Emotional, physical, and sexual abuse in fibromyalgia syndrome: a systematic review with meta-analysis	J GEN INTERN MED	2003	198
10	HÄUSER W	The long-term health outcomes of childhood abuse. An overview and a call to action	ARTHRIT CARE RES	2011	192

Ranked by total local citations.

**Table 3:** Top 10 most locally cited publications.

No.	First author	Article title	Journal	Year	Local citations
1	DUBE SR	Cumulative childhood stress and autoimmune diseases in adults	PSYCHOSOM MED	2009	48
2	SPITZER C	Childhood trauma in multiple sclerosis: a case-control study	PSYCHOSOM MED	2012	23
3	WALKER EA	Psychosocial factors in fibromyalgia compared with rheumatoid arthritis: II. Sexual, physical, and emotional abuse and neglect	PSYCHOSOM MED	1997	17
4	SPITZER C	Gender-specific association between childhood trauma and rheumatoid arthritis: a case-control study	PSYCHOSOM RES	2013	15
5	GOODWIN RD	Association between childhood trauma and physical disorders among adults in the United States	PSYCHOL MED	2004	14
6	DEQUATTRO K	Relationships Between Adverse Childhood Experiences and Health Status in Systemic Lupus Erythematosus	CARE RES	2020	14
7	SHAW MT	Adverse Childhood Experiences Are Linked to Age of Onset and Reading Recognition in Multiple Sclerosis	FRONT NEUROL	2017	13
8	FELDMAN CH	Association of Childhood Abuse with Incident Systemic Lupus Erythematosus in Adulthood in a Longitudinal Cohort of Women	J RHEUMATOL	2019	12
9	BOISSETPIORO MH	Sexual and physical abuse in women with fibromyalgia syndrome	ARTHRITIS RHEUM	1995	11
10	ROBERTS AL	Association of Trauma and Posttraumatic Stress Disorder With Incident Systemic Lupus Erythematosus in a Longitudinal Cohort of Women	ARTHRITIS RHEUMATOL	2017	11

Ranked by total local citations.

In terms of local citation performance, the top-ranked publications more directly constituted the core knowledge network within the field of childhood adversity and autoimmune diseases. Among them, Dube SR’s 2009 paper *Cumulative childhood stress and autoimmune diseases in adults* had the highest local citation count (48), indicating its particularly prominent hub role within the field (30). Spitzer C’s case-control studies on childhood trauma and multiple sclerosis, as well as on gender differences in the association between childhood trauma and rheumatoid arthritis, also ranked highly, suggesting that multiple sclerosis and rheumatoid arthritis are major disease foci within this research topic (31). Meanwhile, studies related to systemic lupus erythematosus, including those by DeQuattro K, Feldman CH, and Roberts AL, also appeared among the top 10 locally cited papers, indicating that the associations between childhood adversity and the occurrence, age at onset, and trauma-related mechanisms of systemic lupus erythematosus have gradually become an important research direction within the field (32–34). Overall, compared with globally highly cited publications, locally highly cited studies are more specifically focused on empirical associations between childhood trauma exposure and particular autoimmune disease types, whereas globally highly cited publications more strongly reflect the broader academic connections of this field with stress biology, psychiatry, and psychoneuroimmunology.

### 3.9 Top 10 most cited journals, authors, and countries/regions in the field of childhood adversity and autoimmune diseases

**Figure 7:**
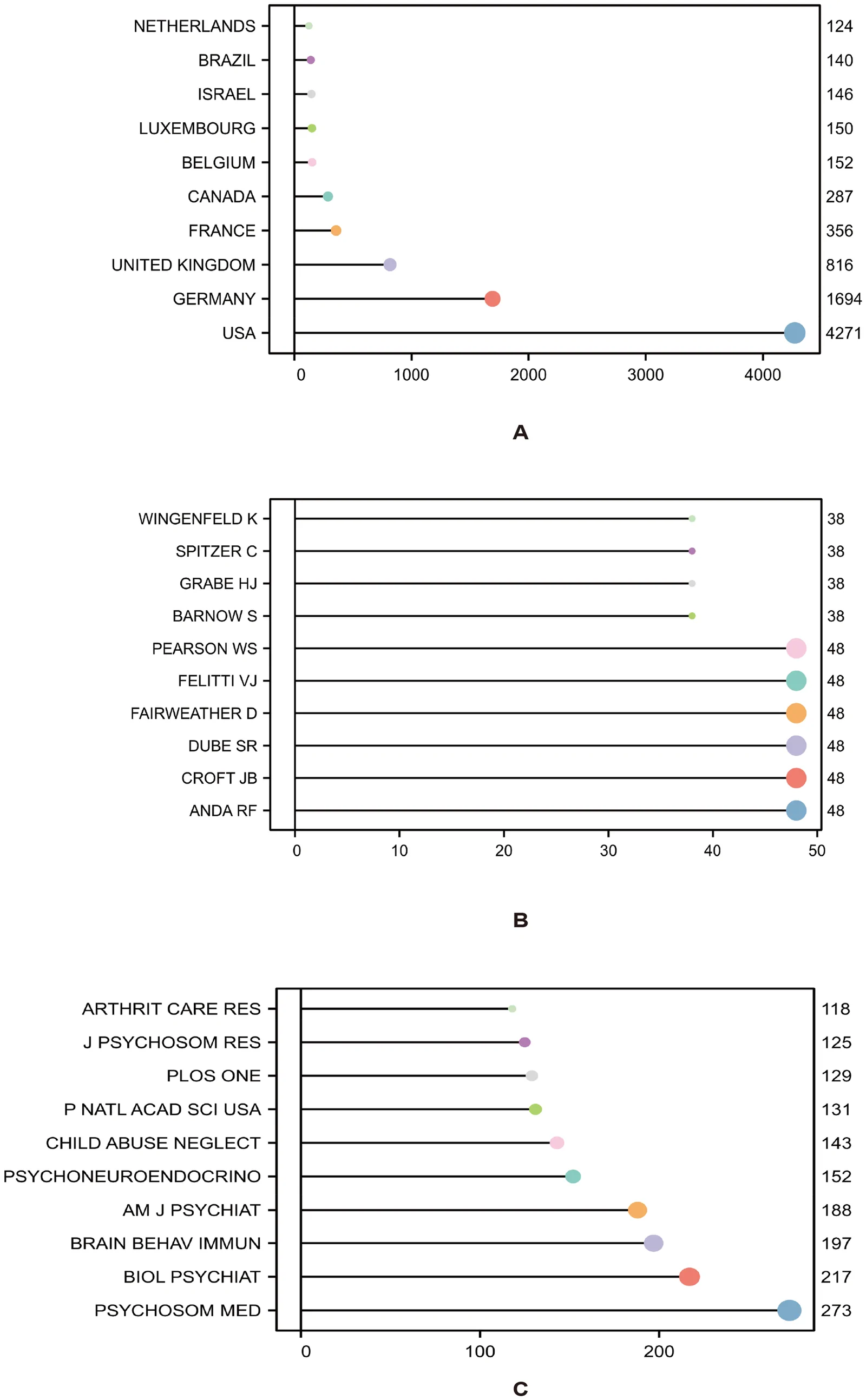
Top 10 countries **(A)**, authors **(B)**, and journals **(C)** by total citations.

**Figure 7** presents the top 10 countries, authors, and journals ranked by total citation counts. At the country level (**Figure 7A**), the United States had the highest total citation count (4,271), far exceeding all other countries, followed by Germany (1,694) and the United Kingdom (816), indicating that these three countries have strong academic influence in this field. At the author level (**Figure 7B**), Anda RF, Croft JB, Dube SR, Fairweather D, Felitti VJ, and Pearson WS shared the highest total citation count (48 each), followed by Barnow S, Grabe HJ, Spitzer C, and Wingenfeld K (38 each). At the journal level (**Figure 7C**), *Psychosomatic Medicine* had the highest total citation count (273), followed by *Biological Psychiatry* (217) and *Brain, Behavior, and Immunity* (197), suggesting that highly influential studies in this field are primarily published in journals related to psychiatry, psychosomatic medicine, and psychoneuroimmunology.

### 3.10 Sensitivity analysis according to document type

An article-only sensitivity analysis was performed to assess the influence of review articles. Eighteen of the top 20 keywords were retained (90.0%; Jaccard similarity = 0.818), and 16 of the top 20 journals were shared between the overall and article-only analyses (80.0%), indicating relatively stable thematic and source-level patterns. In contrast, only 11 of the top 20 most-cited publications were retained in the article-only ranking (55.0%). Reviews accounted for 9 of the overall top 20 most-cited publications (45.0%) despite representing 19.9% of the WoSCC article-and-review corpus, indicating a greater influence of reviews on citation-based rankings (**Supplementary Table 2**).

## 4 Discussion

### 4.1 Overview of the main findings

Based on the Web of Science Core Collection (WoSCC), Scopus, and PubMed, this study conducted a systematic bibliometric analysis of research on childhood adversity and autoimmune diseases, providing a relatively comprehensive overview of the publication trajectory, leading countries and institutions, major journals, knowledge base, and research hotspots in this field. Overall, the annual number of publications showed a sustained upward trend. Early studies were relatively scarce, followed by a gradual transition to a stable growth phase after 2009. Growth accelerated further after 2021, with the annual publication peak reached in 2022. These findings suggest that the association between childhood adversity and autoimmune diseases has increasingly become an important interdisciplinary topic spanning psychosomatic medicine, psychoneuroimmunology, rheumatology, immunology, and public health.

Sensitivity analyses further showed that the major thematic and source-level patterns were largely robust to the exclusion of reviews, with 18 of the top 20 keywords and 16 of the top 20 journals retained in the article-only analysis. However, reviews were disproportionately represented among highly cited publications, indicating that document type had a greater influence on citation-based rankings than on the overall thematic structure of the field.

From the perspective of country and regional distribution, the United States showed a clear advantage in publication output, citation impact, and international collaboration, serving as both the major center of knowledge production and a key hub for international cooperation in this field. The United Kingdom, Canada, Germany, and Australia also demonstrated strong research activity and a certain degree of international influence. Notably, countries such as Australia, Denmark, and Switzerland had relatively high proportions of multiple-country publications, indicating that international collaboration has become an important mode of knowledge production in this field. At the institutional level, leading contributors were mainly concentrated in major North American and European universities and medical institutions. At the journal level, relevant studies were primarily published in *Journal of Psychosomatic Research*, *Psychosomatic Medicine*, *Psychoneuroendocrinology*, and rheumatology- and immunology-related journals. This distribution pattern indicates that the field has developed a relatively clear interdisciplinary research structure, rather than remaining confined to a single clinical specialty.

### 4.2 Knowledge base and research hotspots

Citation analysis further revealed the knowledge base of this field. Globally highly cited publications were mainly concentrated on topics such as stress biology, inflammatory responses, depressive disorders, and the long-term health consequences of childhood trauma, suggesting that psychosocial stress theory and psychoneuroimmunological mechanisms constitute important theoretical foundations for this area of research. Among them, Slavich’s theoretical work on “from stress to inflammation and major depressive disorder,” together with Heim’s study on hypocortisolism and stress-related bodily disorders, both had substantial global influence (28, 29). This indicates that current understanding of the relationship between childhood adversity and autoimmune diseases has moved beyond simple epidemiological descriptions of association and is increasingly being framed within a more integrative”stress–neuroendocrine–inflammation–disease” model (10). Previous studies have further suggested that childhood adversity may exert long-term effects on health through a process of “biological embedding,” whereby early adverse exposures alter key biological systems responsible for maintaining physiological homeostasis and thereby shape disease susceptibility across the life course. Compared with non-maltreated individuals, those exposed to childhood maltreatment show persistent alterations in neural, endocrine, and immune systems in both childhood and adulthood, mainly manifested as structural changes in brain regions such as the prefrontal cortex and hippocampus, enhanced activation of the hypothalamic-pituitary-adrenal (HPA) axis, and elevated levels of inflammation (35). Although some of the currently available evidence is derived primarily from cross-sectional studies and is therefore insufficient to fully support strict longitudinal causal inference, the overall findings still suggest that childhood adversity may induce sustained biological alterations early in life, impairing the maturation and functional balance of homeostatic systems. Such chronic activation may then accumulate into allostatic load and overload, ultimately exerting profound effects on biological aging and long-term health outcomes (36, 37).

Compared with globally highly cited publications, locally highly cited papers may better reflect the internal core knowledge structure of this research topic. Dube et al.’s study on cumulative childhood stress and autoimmune diseases in adults ranked first in local citations, highlighting its foundational and central role within the field (30). This finding suggests that, although theoretical interpretations in this area have drawn extensively on external knowledge resources such as stress biology, life-course epidemiology, and psychoneuroimmunology, the studies that have truly driven the field forward are those directly focused on the association between childhood adversity and autoimmune diseases (17). The strong local influence of Dube et al.’s work may lie in the fact that it was among the earlier studies to directly link cumulative childhood adversity exposure with the risk of autoimmune diseases in adulthood, thereby not only providing a clear research direction for subsequent studies but also shifting the field from broad discussions of health risk toward a more focused analysis of immune-related outcomes.

This line of inquiry was further extended in later studies. Using a nationwide Danish birth cohort, Malham et al. moved the analytical focus earlier in the life course and systematically examined the relationship between multidimensional adversity exposure during the first 1,000 days after birth and the risk of pediatric immune-mediated inflammatory diseases (pIMIDs). Their findings showed that cumulative early biological adversity was significantly associated with an increased risk of pIMIDs and exhibited a dose-response pattern to some extent, while machine-learning analyses further suggested that specific combinations of adversity exposures could substantially increase disease risk in certain subgroups. The importance of this study lies in its extension of the link between childhood adversity and immune-related diseases from retrospective adult associations to prospective risk identification in childhood and within a life-course framework, indicating that the impact of adversity exposure on immune-related diseases may begin as early as the first stages of life (38). Taken together, from Dube et al.’s work on adult autoimmune disease risk to Malham et al.’s birth cohort analysis of pediatric immune-mediated inflammatory diseases, the field appears to be moving from broad associations between childhood adversity and adult health toward more in-depth investigation of specific immune-related diseases, distinct life stages, and particular exposure patterns. This shift indicates that the research focus has progressed from the initial question of “whether an association exists” to more targeted questions, such as “when do the effects begin,” “for which diseases are they more pronounced,” and “which combinations of adversity and pathways are most important,” suggesting that future research is likely to continue developing along lines of disease specificity, life-stage refinement, and deeper mechanistic explanation.

Several other highly locally cited studies have focused on diseases such as multiple sclerosis, rheumatoid arthritis, fibromyalgia, and systemic lupus erythematosus, further indicating that research in this field is shifting from broad associations between childhood adversity and adult health toward more focused investigations of specific autoimmune or immune-inflammatory diseases. Taking multiple sclerosis as an example, the systematic review by Carri S. Polick et al. found that, although the current number of studies remains limited and substantial heterogeneity exists in the measurement of adversity exposure, control of confounding factors, and disease classification, most included studies still suggested an association between childhood adversity and either the risk of multiple sclerosis or its clinical manifestations, including age at onset, relapse, pain, fatigue, and disability (39). These findings imply that the impact of childhood adversity on immune-related diseases may not be limited to disease onset, but may also extend to disease activity, symptom burden, and functional impairment.

Similarly, using data from the 2023 U.S. Behavioral Risk Factor Surveillance System (BRFSS), Qinxin Zhou et al. found that adverse childhood experiences were significantly associated with arthritis risk in adulthood and showed a clear dose-response relationship: compared with individuals without ACEs, those exposed to four or more ACEs had a 55% higher risk of arthritis. Further mediation analyses showed that smoking and depression each explained part of the total effect, suggesting that childhood adversity may influence disease risk not through a single biological pathway alone, but through the joint action of behavioral, psychological, and physiological mechanisms. From a population epidemiological perspective, this study further supports the potential link between childhood adversity and rheumatic and immune-related diseases, while also highlighting the need to consider both health behaviors and psychological factors as mediators of increased disease risk (40). At the same time, studies on fibromyalgia suggest that childhood trauma and other early adverse experiences may be associated with the development of fibromyalgia, pain sensitization, and greater symptom burden in adulthood. Patrick Olivieri et al. proposed that adverse childhood experiences may represent an important early-life risk factor for fibromyalgia, indicating that the impact of childhood adversity may extend beyond typical autoimmune diseases to a broader disease spectrum closely related to immune inflammation, stress dysregulation, and chronic pain (41). For systemic lupus erythematosus, a cross-sectional study by Elisa Esteves Rossini et al. showed that adverse childhood experiences were significantly associated with certain clinical manifestations in adult women with SLE. Specifically, different types of childhood trauma were associated with clinical features such as oral ulcers, nephritis, Sjögren syndrome, photosensitivity, and diabetes. These findings suggest that early adversity exposure may not only contribute to disease susceptibility, but may also influence symptom expression and clinical heterogeneity, thereby increasing the complexity of subsequent disease management (42). Overall, these studies collectively indicate that the association between childhood adversity and immune-related diseases is characterized by substantial disease specificity and clinical complexity. The focus of this field has gradually shifted from broad descriptions of association toward mechanistic interpretation and clinical translation, thereby laying the foundation for future research on disease subtyping, risk stratification, and individualized intervention.

The results of the keyword analysis were broadly consistent with these observations. High-frequency keywords included “childhood adversity,” “depression,” “multiple sclerosis,” “rheumatoid arthritis,” “stress,” “autoimmune disease,” “inflammation,” “anxiety,” “posttraumatic stress disorder,” and “abuse,” indicating that the field currently revolves around three interrelated research themes: first, childhood adversity exposure itself and its subtypes; second, psychological and psychiatric problems such as depression, anxiety, and post-traumatic stress disorder; and third, inflammation, autoimmune diseases, and specific disease subtypes. This keyword structure suggests that the field is not developing along a simple linear “exposure–disease” pathway, but is instead gradually forming a multi-layered research framework encompassing psychosocial exposures, psychological responses, and immune-inflammatory outcomes. In other words, childhood adversity functions in this field both as an initiating exposure and as an upstream background factor linking psychological symptoms to physical disease outcomes.

More specifically, the co-occurrence of childhood adversity-related terms with mechanistic and disease-related keywords such as “stress,” “inflammation,” and “autoimmune disease” indicates that the field has gradually moved from early identification and classification of exposures toward a more integrated explanation of biological embedding processes and disease consequences. In particular, the appearance of specific disease terms such as “multiple sclerosis,” “rheumatoid arthritis,” and “systemic lupus erythematosus” reflects a shift from broad chronic disease and general adverse health outcomes toward specific clinical contexts of immune-mediated diseases. This change suggests that researchers are paying increasing attention to differences among disease types in terms of pathogenesis, clinical presentation, and stress sensitivity, and also implies that future research needs to further examine the pathways linking childhood adversity to disease within disease-specific frameworks.

The review by Michelle A. Chen et al. further supports this interpretation. That study emphasized that childhood adversity is closely associated with a wide range of mental and physical health problems across the lifespan, and that individuals exposed to childhood abuse, neglect, family conflict, poor parent-child relationships, low socioeconomic status, or extreme poverty face higher risks of illness and premature mortality than unexposed individuals. More importantly, from a psychoneuroimmunological perspective, the review systematically summarized the links between childhood adversity and immune dysregulation, indicating that early-life stress may be associated with increased proinflammatory cytokine production, heightened susceptibility to infection and disease, reactivation of latent herpesviruses, impaired antitumor immune responses, and alterations in epigenetic processes (11). Janusek et al.’s study of African American young men provided more direct mechanistic evidence. They found that greater childhood trauma and indirect exposure to neighborhood violence were associated with a stronger interleukin-6 (IL-6) inflammatory response to acute psychosocial stress, together with a relatively blunted cortisol response; in addition, lower methylation of the IL-6 promoter was associated with higher childhood trauma exposure and stronger stress-induced IL-6 responses (43). These findings suggest that the effects of childhood adversity on disease risk are not confined to a single psychological or behavioral pathway, but may instead continuously shape disease susceptibility across the life course through multiple biological mechanisms, including immune dysregulation and epigenetic regulation, thereby further supporting a pathway of “early adversity–epigenetic remodeling–amplified inflammation–increased disease susceptibility.

Notably, mental health-related terms such as “depression,” “anxiety,” and “posttraumatic stress disorder” were closely linked in the co-word network to disease- and mechanism-related terms such as “inflammation,” “multiple sclerosis,” “rheumatoid arthritis,” and “systemic lupus erythematosus.” This suggests that psychological disorders may not merely represent coexisting outcomes in this field, but may instead serve as mediating, moderating, or amplifying factors in the process through which childhood adversity affects immune function and disease risk. On the one hand, childhood adversity may increase chronic stress burden by elevating depressive, anxiety, and post-traumatic stress symptoms, thereby influencing immune homeostasis through neuroendocrine and inflammatory pathways (44, 45).On the other hand, psychological problems themselves may indirectly affect disease onset, symptom burden, and prognosis through unhealthy behaviors, sleep disturbance, reduced treatment adherence, and impaired social functioning (28, 46). Therefore, psychological problems and immune-related diseases in this field are more likely to be intertwined and mutually reinforcing rather than representing two fully independent outcome domains.

From the perspective of research development, this keyword co-occurrence pattern also suggests that the field is shifting from a simple descriptive model of “childhood adversity–adult disease” toward a chained explanatory framework of “childhood adversity–psychological stress/psychiatric disorders–immune-inflammatory abnormalities–disease outcomes.” This not only reflects strong interdisciplinary integration, but also suggests that future studies should place greater emphasis on multifactorial, multipathway, and multilevel analytical approaches. For example, future study designs could integrate longitudinal cohorts, symptom trajectory analyses, biomarker assessments, and mediation models to identify the specific roles played by psychological factors in the relationship between childhood adversity and autoimmune diseases. At the clinical and public health levels, greater attention should also be paid to the potential value of trauma-informed care, psychological screening, and early intervention in the management of immune-related diseases.

### 4.3 Evolution of thematic structure and interdisciplinary characteristics

According to the clustering results generated by VOSviewer and CiteSpace, the research themes in this field show clear aggregation and evolution. Over the past decade, keyword clusters have focused more on topics such as post-traumatic stress disorder, cumulative childhood stress, the hypothalamic-pituitary-adrenal axis, depressive symptoms, and systemic lupus erythematosus, indicating that recent studies have paid increasing attention to the biological mechanisms of psychological stress and the effects of trauma exposure within specific disease contexts. By contrast, the clustering results covering the entire study period up to 2025 encompassed a broader range of themes, including classic exposure concepts such as “adverse childhood experiences,” “childhood maltreatment,” and “sexual abuse,” as well as disease-, symptom-, and mechanism-related topics such as “systemic lupus erythematosus,” “posttraumatic stress disorder,” “sex difference,” and “c-reactive protein.” These findings suggest that the knowledge structure of this field has evolved from conceptual construction and exploratory association studies toward increasing emphasis on disease specificity, sex differences, and biomarker-based explanations.

In addition, the distribution of journals and the journal density map also reflect the strongly interdisciplinary nature of this field. High-density journals were mainly concentrated in psychosomatic medicine, psychiatry, neuroendocrinology, rheumatology, and immunology, indicating that research on childhood adversity and autoimmune diseases is not simply a subtopic of a single disease-based specialty, but rather a comprehensive issue that connects early-life exposures, psychological stress responses, neuroendocrine regulation, immune-inflammatory abnormalities, and the onset and progression of chronic diseases. On the one hand, this interdisciplinary feature broadens the research perspective; on the other hand, it also suggests that future studies will require more standardized definitions, unified measurement tools, and integrative research designs to improve comparability and synthesis across disciplinary boundaries (47–49).

### 4.4 Clinical and public health implications

The findings of this study suggest that childhood adversity may represent an important early life-course exposure that should not be overlooked in research on autoimmune diseases. Both previously highly cited studies and current research hotspots indicate that childhood adversity is closely associated not only with psychological disorders in adulthood, but also potentially with the onset, disease activity, symptom burden, and prognosis of autoimmune diseases through pathways involving chronic stress load, HPA-axis dysregulation, inflammatory activation, and immune dysfunction. Therefore, in clinical practice, appropriate attention to histories of early adverse experiences and trauma exposure among patients with diseases such as rheumatoid arthritis, multiple sclerosis, systemic lupus erythematosus, and fibromyalgia may help to provide a more comprehensive understanding of symptom heterogeneity, psychiatric comorbidity, and long-term disease management needs (17, 39, 41).

From a public health perspective, the current body of research also supports the inclusion of childhood adversity within life-course prevention frameworks for chronic and immune-related diseases. Traditional chronic disease prevention has largely focused on adult lifestyle factors and biomedical risk factors, whereas the present findings suggest that psychosocial exposures early in life may exert long-lasting and profound influences on disease risk in adulthood. Accordingly, strengthening child protection, reducing childhood exposure to violence and abuse, and improving early psychological intervention and family support may have significance not only for child welfare and mental health, but also for the long-term prevention of immune-related diseases and the promotion of health equity (50).

### 4.5 Strengths and limitations

This study has several strengths. First, by incorporating the WoSCC, Scopus, and PubMed databases, it achieved broader literature coverage and thus enabled a more comprehensive depiction of the research landscape in this field. Second, the study adopted a two-step deduplication strategy of “DOI first, title-assisted second,” and further cleaned and standardized information such as country names and keywords, thereby improving the reliability and interpretability of the results. Third, by combining annual trend analysis, country and institutional collaboration analysis, highly cited literature analysis, keyword co-occurrence analysis, and CiteSpace clustering, this study revealed the knowledge structure and evolutionary trajectory of the field from multiple dimensions.

Nevertheless, several limitations should also be acknowledged. First, although a multi-database integration strategy was used, differences remain among databases in terms of coverage, citation structure, and indexing rules, and some statistical results may therefore still be affected by database heterogeneity. Second, citation relationship analyses were conducted primarily on the basis of WoSCC data; although this helps ensure consistency in citation structure, it may underestimate the influence of some studies not fully covered by WoSCC. Third, the search strategy itself inevitably shaped the scope of the retrieved literature and, consequently, the observed keyword distributions, cluster structures, and thematic evolution. Because the exposure model and outcome model were constructed using predefined terms related to childhood adversity and autoimmune diseases, the results were limited to the concepts captured by these search terms. Therefore, less frequently used terms, rare forms of childhood stressors, or relatively uncommon autoimmune diseases may have been underrepresented or omitted. Fourth, although keyword cleaning and synonym merging improved the readability of the analyses, these procedures inevitably involved some degree of researcher judgment and may have influenced the presentation of certain marginal themes. Finally, bibliometric analysis essentially reflects patterns of publication and citation behavior; while it can reveal knowledge structures and research hotspots, it cannot directly replace original epidemiological evidence in determining the causal relationship between childhood adversity and autoimmune diseases (51). Therefore, mechanistic interpretations and clinical inferences in this field still need to be further validated by high-quality longitudinal studies, causal inference research, and mechanistic experimental studies.

In addition, original articles and reviews were analyzed together in the primary bibliometric mapping. Although the article-only sensitivity analysis showed high concordance in major keywords and journal rankings, reviews accounted for 45.0% of the top 20 most-cited publications while representing 19.9% of the WoSCC article-and-review corpus. Therefore, citation-based rankings should be interpreted with consideration of the greater citation influence of review articles.

In summary, research on childhood adversity and autoimmune diseases has shown sustained growth in recent years and has developed into an interdisciplinary research landscape characterized by U.S. leadership, broad multinational participation, and clear cross-disciplinary integration. Current research hotspots are mainly concentrated on mental health problems such as depression, anxiety, and post-traumatic stress disorder, as well as inflammatory responses, multiple sclerosis, rheumatoid arthritis, systemic lupus erythematosus, and other immune-related diseases and mechanistic topics. Highly cited literature and clustering results together indicate that the field is gradually moving from early descriptive association studies toward a more integrative explanatory framework centered on early-life stress, biological embedding, neuroendocrine dysregulation, and immune-inflammatory abnormalities. Future research should further strengthen international collaboration, promote the standardization of exposure definitions, measurement tools, and study designs, and conduct more longitudinal and mechanistic studies to better elucidate the long-term impact of childhood adversity on the onset, progression, and prognosis of autoimmune diseases.

## 5 Conclusion

Based on three major databases, this study conducted a systematic bibliometric analysis of research on childhood adversity and autoimmune diseases. The results show that the number of publications in this field has continued to increase in recent years, forming an international research landscape centered on the United States and jointly shaped by countries such as the United Kingdom, Canada, Germany, and Australia. Core research forces are mainly concentrated in North America and Europe, and the related publications are primarily distributed in journals of psychosomatic medicine, psychoneuroendocrinology, and rheumatology/immunology, indicating the distinctly interdisciplinary nature of this topic. Keyword co-occurrence, clustering, and citation analyses further suggest that the field has gradually evolved from early descriptive studies of the relationship between childhood adversity and adult health risk toward more mechanistic and disease-specific investigations focused on inflammatory responses, HPA-axis dysregulation, psychiatric comorbidity, and specific autoimmune disease outcomes. Overall, childhood adversity may represent an important long-term risk factor for the development and progression of autoimmune diseases. Future research should further promote standardized measurement, expand longitudinal follow-up and causal inference studies, and facilitate the translation of mechanistic findings into risk identification, precision prevention, and clinical intervention.

Sensitivity analyses supported the robustness of the major thematic and source-level findings after exclusion of reviews, although review articles exerted a greater influence on citation-based rankings.

## Data Availability

The original contributions presented in the study are included in the article/**Supplementary Material**. Further inquiries can be directed to the corresponding author.
