# Supplementary material for: Childhood adversity and autoimmune diseases: global research landscape, immune-related hotspots, and emerging trends from a bibliometric analysis of three databases
Source: Front Immunol. 2026 Aug 31;17:1864301. doi: 10.3389/fimmu.2026.1864301 (PMC13572917; doi:10.3389/fimmu.2026.1864301)
Supplement: Supplementary file 1 [file Table1.docx]

**Supplementary Table 1. Search strategies for WoSCC, Scopus, and PubMed**

**Web of Science Core Collection (WoSCC)**

| **Item** | **Content** |
| --- | --- |
| Search strategy | TS=(  "adverse childhood experiences"  OR "childhood adversity"  OR "early life adversity"  OR "childhood trauma"  OR "childhood maltreatment"  OR "childhood sexual abuse"  OR "childhood physical abuse"  OR "childhood emotional abuse"  OR "childhood neglect"  OR "child abuse"  OR "child neglect"  )  AND  TS=(  "autoimmune disease*"  OR "autoimmune disorder*"  OR "rheumatoid arthritis"  OR "systemic lupus erythematosus"  OR "multiple sclerosis"  OR "type 1 diabetes"  OR "autoimmune thyroiditis"  OR "Hashimoto thyroiditis"  OR "Graves disease"  OR "Sjogren syndrome"  OR "celiac disease"  ) |
| Publication years | Database inception to December 31, 2025 |
| Document types | Article or Review Article |
| Language | English |

**Scopus**

| **Item** | **Content** |
| --- | --- |
| Search strategy | TITLE-ABS-KEY(  "adverse childhood experiences"  OR "childhood adversity"  OR "early life adversity"  OR "childhood trauma"  OR "childhood maltreatment"  OR "childhood sexual abuse"  OR "childhood physical abuse"  OR "childhood emotional abuse"  OR "childhood neglect"  OR "child abuse"  OR "child neglect"  )  AND  TITLE-ABS-KEY(  "autoimmune disease*"  OR "autoimmune disorder*"  OR "rheumatoid arthritis"  OR "systemic lupus erythematosus"  OR "multiple sclerosis"  OR "type 1 diabetes"  OR "autoimmune thyroiditis"  OR "Hashimoto thyroiditis"  OR "Graves disease"  OR "Sjogren syndrome"  OR "celiac disease"  ) |
| Publication years | Database inception to December 31, 2025 |
| Document types | Article or Review |
| Language | English |

**PubMed**

| **Item** | **Content** |
| --- | --- |
| Search strategy | (  "adverse childhood experiences"[Title/Abstract]  OR "childhood adversity"[Title/Abstract]  OR "early life adversity"[Title/Abstract]  OR "childhood trauma"[Title/Abstract]  OR "childhood maltreatment"[Title/Abstract]  OR "childhood sexual abuse"[Title/Abstract]  OR "childhood physical abuse"[Title/Abstract]  OR "childhood emotional abuse"[Title/Abstract]  OR "childhood neglect"[Title/Abstract]  OR "child abuse"[Title/Abstract]  OR "child neglect"[Title/Abstract]  )  AND  (  "autoimmune disease*"[Title/Abstract]  OR "autoimmune disorder*"[Title/Abstract]  OR "rheumatoid arthritis"[Title/Abstract]  OR "systemic lupus erythematosus"[Title/Abstract]  OR "multiple sclerosis"[Title/Abstract]  OR "type 1 diabetes"[Title/Abstract]  OR "autoimmune thyroiditis"[Title/Abstract]  OR "Hashimoto thyroiditis"[Title/Abstract]  OR "Graves disease"[Title/Abstract]  OR "Sjogren syndrome"[Title/Abstract]  OR "celiac disease"[Title/Abstract]  ) |
| Publication years | Database inception to December 31, 2025 |
| Language | English |

**Supplementary Table 2. Document-type distribution and sensitivity analysis**

| **Category** | **Indicator** | **Result** |
| --- | --- | --- |
| Document-type distribution | Original articles | 257/319 (80.6%) |
|  | Reviews | 62/319 (19.4%) |
| Sensitivity analysis | Shared Top 20 keywords, overall vs. article-only | 18/20 (90.0%) |
|  | Jaccard similarity of Top 20 keywords | 0.818 |
|  | Shared Top 20 journals, overall vs. article-only | 16/20 (80.0%) |
|  | Shared Top 20 most-cited publications, overall vs. article-only | 11/20 (55.0%) |
|  | Reviews among WoSCC Top 20 most-cited publications | 9/20 (45.0%) |
|  | Reviews in the WoSCC Article + Review corpus | 19.9% |

**Note:** WoSCC, Web of Science Core Collection. The article-only sensitivity analysis was compared with the overall Article + Review dataset.
